# Use of Whole-Genome Sequencing in the Investigation of a Nosocomial Influenza Virus Outbreak

**DOI:** 10.1093/infdis/jiy335

**Published:** 2018-06-05

**Authors:** Catherine F Houlihan, Dan Frampton, R Bridget Ferns, Jade Raffle, Paul Grant, Myriam Reidy, Leila Hail, Kirsty Thomson, Frank Mattes, Zisis Kozlakidis, Deenan Pillay, Andrew Hayward, Eleni Nastouli

**Affiliations:** 1 Division of Infection and Immunity, University College London, London, United Kingdom; 2 Institute of Epidemiology and Health Care, University College London, London, United Kingdom; 3 Department of Population, Policy, and Practice, Great Ormond Street Institute of Child Health, University College London (UCL), London, United Kingdom; 4 National Institute for Health Research Biomedical Research Centre, London, United Kingdom; 5 Department of Clinical Virology, UCL Hospitals National Health Service Foundation Trust, London, United Kingdom; 6 Infection Control Service, UCL Hospitals National Health Service Foundation Trust, London, United Kingdom; 7 Department of Blood Diseases, UCL Hospitals National Health Service Foundation Trust, London, United Kingdom; 8 Department of Infectious Disease Informatics, Farr Institute of Health Informatics Research, London, United Kingdom

**Keywords:** Influenza, nosocomial, sequencing, transmission

## Abstract

Traditional epidemiological investigation of nosocomial transmission of influenza involves the identification of patients who have the same influenza virus type and who have overlapped in time and place. This method may misidentify transmission where it has not occurred or miss transmission when it has. We used influenza virus whole-genome sequencing (WGS) to investigate an outbreak of influenza A virus infection in a hematology/oncology ward and identified 2 separate introductions, one of which resulted in 5 additional infections and 79 bed-days lost. Results from WGS are becoming rapidly available and may supplement traditional infection control procedures in the investigation and management of nosocomial outbreaks.

## Teaser

Influenza virus whole-genome sequencing (WGS) was used to investigate an outbreak of influenza A virus infection in a hematology/oncology ward; 2 separate introductions were identified. The role of WGS for nosocomial outbreak investigation is discussed.

Nosocomial transmission of influenza A virus is of significant concern since infection in individuals who are immunocompromised, immunosuppressed, at extremes of age, or pregnant have an increased risk of severe illness, morbidity and death [1, 2]. The risk of nosocomial acquisition of influenza virus is high since patients commonly share open bays before viral respiratory infection is diagnosed, and significant healthcare costs are associated with hospital ward closures due to influenza virus outbreaks. Influenza can also be asymptomatic, and prolonged shedding has been demonstrated in those who are immunocompromised [3]. Low rates of influenza vaccination in healthcare staff in England (49.5% in January 2016) combined with reduced vaccine efficacy raises the possibility that healthcare staff are involved in nosocomial transmission [2].

Commonly, nosocomial transmission of influenza virus within the healthcare setting has been identified through traditional molecular diagnostic methods (ie, real-time polymerase chain reaction [PCR] analysis and reverse transcription PCR [RT-PCR]) for the detection of viral species, combined with data collected on patient and staff movement within the hospital. Patients who have overlapped in time and space and who have evidence of infection with the same viral type and, if available, subtype are assumed to have transmitted to each other. Direct Sanger sequencing methods have more recently been used to identify possible clusters of nosocomial influenza virus infection [4], but next-generation sequencing (NGS) of the whole virus genome, which may offer increased discriminatory capacity, has been infrequently reported. We describe an outbreak of influenza A virus infection, during early 2016, on a hematology/oncology ward in a National Health Service (NHS) hospital in London, in which timely use of whole-genome sequencing (WGS) would have identified the presence or absence of nosocomial transmission and allowed a more targeted infection control response.

## CLINICAL CASES

**Figure 1.**
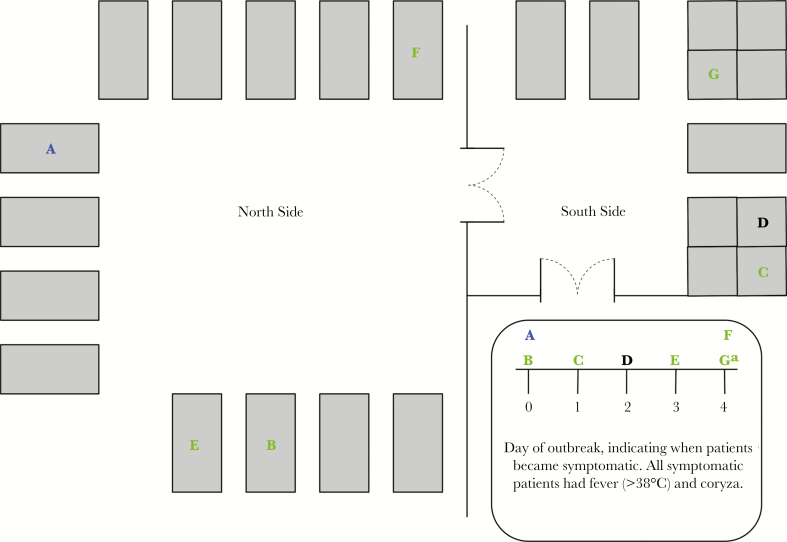
Map of the ward. Rectangles are side rooms, and squares indicate bays with 4 beds. Letters indicate patients and are colored green or purple according to whether influenza virus sequences are considered part of the same transmission cluster. Patient D is colored black since the genome coverage was considered of sufficient depth. ^a^Patient G was asymptomatic but tested positive for influenza virus A on day 4.

Two patients with hematological/oncological malignancies (patients A and B; Figure 1), who had each been admitted for 2 and 5 weeks, respectively, on the north side of ward 1, a hematology/oncology ward in a London NHS hospital, developed coryzal symptoms with fever in the same 24-hour period. Combined nose and throat swab specimens were collected from each patient at symptom onset (day 0), as per the hospital’s policy, and were tested using multiplex RT-PCR analysis involving a standard panel of 6 respiratory viruses, including influenza A and B viruses. Results were available on day 1, and influenza A virus was detected in samples from both patients. Nosocomial transmission was not assumed to have occurred between these patients, since they were in separate positive-pressure rooms. The mechanism was therefore uncertain. On day 1, a further patient (patient C; Figure 1) on the south side of the ward, developed symptoms and tested positive for influenza A virus. Since this south-side patient had no clear link to patients A and B on the north side, it was assumed that an outbreak was not occurring, and the ward was not closed. Over the next 48 hours, a further 2 patients (patients D and E) tested positive for influenza A virus (on days 2 and 3, respectively), and the ward was closed on day 3. Testing of 1 further symptomatic patient and screening of all asymptomatic patients on day 4 identified 2 further patients (patient F, who was symptomatic, and patient G, who was asymptomatic). A visiting relative of patient B later revealed that they had coryzal symptoms that preceded and continued throughout the outbreak. This relative tested positive for influenza A virus 1 week after the first patients were tested.

A total of 7 patients developed influenza A virus infection on this ward. Of these, patient E required a prolonged period in intensive care. Thirteen patients who were contacts of the patients described above were screened, had negative test results, and commenced prophylaxis, in keeping with Public Health England guidance and the NHS trust policy [5]. The ward was closed for 6 days and resulted in 79 bed-days lost. Five healthcare workers developed influenza-like illness and were not permitted to attend work, as per the NHS trust’s occupational health policy. They were not tested for influenza virus.

## MATERIALS AND METHODS

Samples from the 7 patients on the same ward, 9 samples that were submitted to the same laboratory on the same days from the same hospital, and a sample from the symptomatic relative who had tested positive for influenza A virus on the basis of standard in-house RT-PCR were sequenced using the Mi-Seq (Illumina) platform.

Amplification of influenza A virus RNA was performed using the SuperScript III One-Step RT-PCR kit (Invitrogen) and a modified 8-segment PCR method [6]. Fifty-microliter reactions containing 10.0 µL of RNA, a final concentration of 1× SuperScript III One-Step RT-PCR buffer, 0.1 mM of each primer, and 1.0 µL of SuperScript III RT/Platinum Taq high-fidelity enzyme were prepared. RT-PCR thermal cycling conditions were as described elsewhere [6]. Two PCR amplicons were generated; equal volumes of both were combined, and the total DNA concentration was determined using the Qubit HS DNA assay (Invitrogen).

Library preparations were generated using the Nextera XT DNA sample preparation kit (Illumina) according to manufacturer’s instructions, using 1 ng of input DNA. Tagged PCR samples were purified with 30 µL of AMPure XP beads (catalog no. A63881; Agencourt). Sample library normalization and MiSeq sample loading were carried out according to the Nextera XT protocols. Pooled normalized samples, including a Phi-X control at a final concentration of 0.1 pM, were loaded onto a Mi-Seq reagent kit V3 600 cycle (Illumina) and sequenced on a MiSeq (Illumina).

Consensus genomes were generated from short reads, using ICONIC’s in-house de novo assembly pipeline, applying a read depth cutoff of ≥20 reads to the final sequences. Phylogenetic analysis was undertaken first by separately aligning each set of segments using MAFFT [7] and then concatenating the coding regions within Aliview [8]. Maximum-likelihood phylogenetic trees were inferred for each alignment, using RAxML [9]. Phylogenies were inferred under a general time-reversible substitution model with rate heterogeneity among sites modeled under a 4-category discrete approximation of a gamma distribution. Branch support was assessed through nonparametric bootstrapping of 1000 pseudoreplicates. Direct linkage of whole virus genomes was considered to have statistical support if the observed number of mutations between them occurred within the 95% confidence interval of the expected number (given the mutation rate, interval between sample collection, genome, and pair-wise alignment lengths), and they clustered with respective bootstrap values ≥95% (Supplementary Methods).

## RESULTS

**Figure 2.**
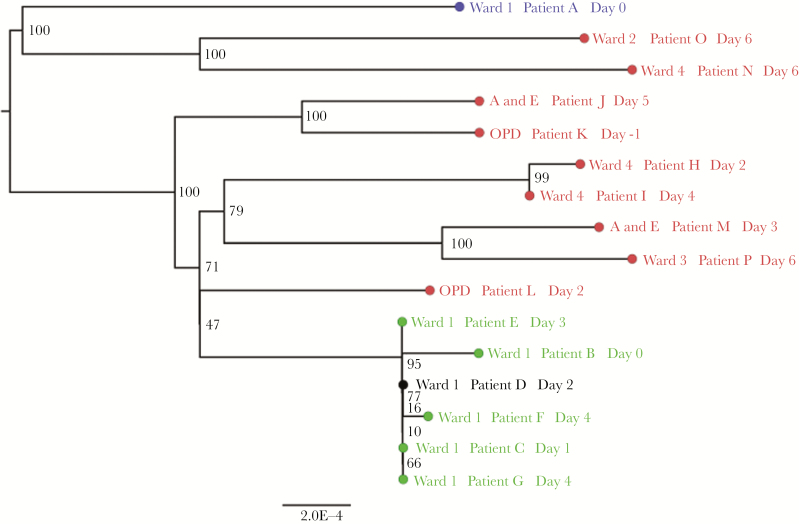
Maximum-likelihood tree derived from a genomic alignment of sequences generated during the influenza outbreak on ward 1. Tips are colored according to location within the hospital and whether the patients are considered part of the same transmission cluster (green, ward 1, linked; blue, ward 1, unlinked; red, elsewhere in the same hospital, unlinked). Tips in black indicate insufficient genome coverage (only the sequence for NS-1 was available). Bootstrap support percentages from 1000 replicates are shown for each node. A and E, Accident and Emergency Department; OPD, Out Patient Department.

Seven samples from 7 hematology/oncology patients, 1 sample from a visiting relative of patient B, and 9 samples from other patients in the same hospital tested positive for influenza A virus, according to multiplex RT-PCR analysis. WGS was successful for 15 of 17 samples, with a median read depth across all samples of 800 (interquartile range, 600–5000). Attempts to sequence the sample from the relative of patient A were unsuccessful. The sample from patient D failed to generate sequence across all 8 segments of the genome with sufficient depth, resulting in a reliable sequence for NS1 alone (segment 8). Despite this limitation, when a reliable sequence from this patient’s sample was included in a maximum-likelihood phylogenetic tree analysis, samples from patients B, C, D, E, F, and G clustered tightly with high bootstrap support (>95%), with almost identical sequences (Figure 2). The same result was obtained when the sample from patient D was excluded from the phylogenetic analysis (Supplementary Figure 1).

There was no evidence of transmission between patient A and patient B, who both had influenza A virus detected by RT-PCR analysis on day 1. These 2 patients were likely infected via separate nosocomial transmissions. Incidentally, a further probable (unrecognized) nosocomial transmission was identified on a separate ward (in patients H and I; Figure 2).

## DISCUSSION

Using WGS, we illustrated the dynamics of an influenza virus outbreak on a hematology/oncology ward, clarifying that 2 patients (patients A and B) who were initially considered to be index cases, based on findings of traditional epidemiological methods, were not linked and that one of these individuals was not involved in the outbreak. The use of traditional epidemiological infection control measures erroneously linked these 2 unrelated infections and missed spread of the infection from the north to the south side of the ward, leading to a delay in infection control action. Had WGS data been available in real time, the cross-ward transmission would have been identified on day 1 or 2, and the ward would have been closed. Identification of the separate source of influenza A virus introduction (ie, patient A) would additionally have led to a separate investigation and instigation of infection control procedures specific to this introduction.

The integration of WGS data with epidemiological analysis in nosocomial infections could identify common pathways of transmission on wards, such as communal areas, bays, shared patient equipment, healthcare staff, or visitors interacting with each other and other patients. Early identification of pathways of transmission could prevent further nosocomial cases of influenza. Interventions would include earlier initiation of ward closure; prophylaxis; emphasis on influenza vaccination of staff and, if they are unwell, treatment; enhanced equipment or room cleaning; and the limiting of visitor-visitor or visitor-patient contact. Prophylaxis has been shown to reduce the risk of symptomatic influenza virus infection in immunocompetent and immunocompromised adults by up to 80% [10]. Staff vaccination rates on this ward were extremely low at the time of the outbreak: 25% and 55% of staff working on the north and south ends, respectively, had received the seasonal influenza vaccine (Figure 1). Analysis of the effectiveness of the World Health Organization–recommended seasonal influenza vaccination against the 2009 pandemic influenza A(H1N1) virus circulating at the time has been reported as 65% overall [11]. Demonstration of the nosocomial outbreak described on this ward led anecdotally to increased vaccine uptake by staff.

We achieved full-length genome sequencing for all but 2 isolates in this case, allowing more-accurate linkage of infections, compared with the accuracy achieved via limited sequencing of the hemagglutinin or neuraminidase genes. One of these 2 failed attempts at sequencing may be attributable to sampling the relative of patient B close to the end of their illness, when the quantity and quality of viral RNA is less robust.

NGS has been used to identify common sources of nosocomial outbreaks of norovirus and methicillin-resistant *Staphylococcus aureus*, but it has been used less frequently to identify transmission of respiratory viruses, including influenza virus [12]. Possible transmission between patients can be identified from relatively small amounts of viral RNA in specimens. However, bioinformatics analysis, equipment, and consumables costs have limited the implementation of this technology into routine clinical care. These limitations, as well as reduction in turnaround times, are improving with time and recognition of the clinical usefulness of NGS. WGS is now available with small, lightweight, and easily transportable devices, such as the MinION (Oxford Nanopore). It is expected that NGS results for recognized pathogens with established pipelines (ie, influenza virus sequencing) will, in the near future, be available 24 hours after sample receipt.

Two large studies from an epidemiological surveillance unit in Germany and a retrospective analysis of nosocomial transmissions in care facilities in Canada have confirmed nosocomial influenza virus transmission where it was thought to have occurred, using WGS [13, 14]. Although it could be argued that confirmation of a suspected transmission has high cost with limited benefit, we have shown that WGS can demonstrate separate introductions and allow tailored infection control approaches. Further, the use of NGS during outbreaks of highly infectious diseases with significant consequences and the sharing of data with public health teams have been instrumental in understanding transmission, highlighted in the recent Ebola and Zika outbreaks.

At this hospital, during the 2016 influenza season, WGS allowed early identification of the predominant circulating influenza A virus subtype as H1N1, rather than H3N2, and led to a local policy change from the recommendation of oseltamivir as first-line treatment to zanamivir. Public Health England altered the national guideline in a similar way, 1 month later [5]. Moving forward, the use of WGS would allow a more timely and accurate shift in treatment recommendation, and individualized antiviral treatment for influenza would be possible.

We have described an outbreak of influenza in a hematology/oncology ward in which real-time use of WGS may have mitigated propagation of the outbreak and certainly would have increased understanding of it. The use of WGS during the influenza season, either for all hospitalized patients or specifically for patients who develop influenza during an inpatient stay, may identify transmission where it was not suspected and allow rapid implementation of infection control procedures.

## Supplementary Data

Supplementary materials are available at *The Journal of Infectious Diseases* online. Consisting of data provided by the authors to benefit the reader, the posted materials are not copyedited and are the sole responsibility of the authors, so questions or comments should be addressed to the corresponding author.

Supplementary Figure Legend

Supplementary Figure 1

Supplementary Methods

## Notes

***Financial support.*** This work was supported by the Health Innovation Challenge Fund T5-344 (Infection Response Through Virus Genomics), a parallel funding partnership between the Department of Health and the Wellcome Trust.

***Potential conflicts of interest***. All authors: No reported conflicts of interest. All authors have submitted the ICMJE Form for Disclosure of Potential Conflicts of Interest. Conflicts that the editors consider relevant to the content of the manuscript have been disclosed.
