# Corrigendum

**DOI:** 10.1093/infdis/jiy681

**Published:** 2018-12-26

“Use of Whole-Genome Sequencing in the Investigation of a Nosocomial Influenza Virus Outbreak” by Houlihan et al. [J Infect Dis 2018; 218(9):1485–89]. The caption of figure 1 states “Patient D is colored black since the genome coverage was considered of sufficient depth”. However, in the main image, patient E is in black. The E in black on the main image should read D. In the small sub-box, patient E is colored black but it should be patient D.

The authors regret the error.

