# Supplementary material for: Use of Whole-Genome Sequencing in the Investigation of a Nosocomial Influenza Virus Outbreak
Source: J Infect Dis. 2018 Jun 5;218(9):1485–9. doi: 10.1093/infdis/jiy335 (PMC6151078; doi:10.1093/infdis/jiy335)
Supplement: Supplementary Figure Legend [file jiy335_suppl_supplementary_appendix_a1.docx]

**Appendix figure A1**

Maximum-likelihood tree derived from a genomic alignment of sequences generated at during the influenza outbreak on Ward 1 excluding Patient D. Tips are coloured according to location within the hospital and whether the patients are considered part of the same transmission cluster (green: Ward 1, linked; blue: Ward 1, unlinked; red: elsewhere in the hospital, unlinked). Bootstrap support values (%) from 1000 replicates are shown for each node.
