# Supplementary figures and images for: Use of Whole-Genome Sequencing in the Investigation of a Nosocomial Influenza Virus Outbreak
Source: J Infect Dis. 2018 Jun 5;218(9):1485–9. doi: 10.1093/infdis/jiy335 (PMC6151078; doi:10.1093/infdis/jiy335)

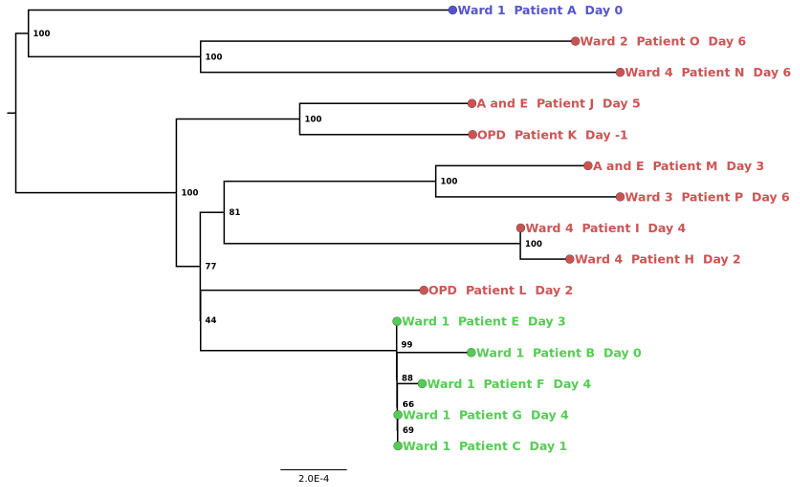

Supplement: Supplementary Figure 1 [file jiy335_suppl_supplementary_figure_a1.png]
