# Supplementary material for: Use of Whole-Genome Sequencing in the Investigation of a Nosocomial Influenza Virus Outbreak
Source: J Infect Dis. 2018 Jun 5;218(9):1485–9. doi: 10.1093/infdis/jiy335 (PMC6151078; doi:10.1093/infdis/jiy335)
Supplement: Supplementary Methods [file jiy335_suppl_supplementary_methods.docx]

**Supplementary Methods**

Calculating the expected number of substitutions between pairs of samples

### When considering the number of differences we expected to see between samples linked in a transmission chain, we first considered all patients involved to be ‘infected’ or incubating (i.e. with a higher replication rate) rather than shedding, since it was an outbreak.

### We used a rough substitution rate estimated from the literature of 5x10^-3^ substitutions per site per year. To put this into perspective, given the flu genome is ~13500 nucleotides long, this corresponds to approximately 68nt per genome per year, or just over 1nt drift per week.

In addition, we used rates of substitution estimated from a separate project, where we had run BEAST analyses of 54 H1N1 sequences sampled at UCLH during two flu seasons (2012-13 and 2013-2014). For all runs, the SDR06 nucleotide substitution model was used, along with a relaxed molecular clock and a Bayesian Skyline coalescent prior. We partitioned the alignment by segments and thus were able to estimate rates of substitution for each segment (and associated variances). Across the entire flu genome this yielded a lower rate of ~1.5x10^-3^ substitutions per site per year (standard deviation ~6.1x10^-4^). From these values, for each pair of samples we were able to calculate a maximum expected number of substitutions using the upper bound for each 95% credibility interval.

We also considered the number of differences we would expect to see due to sequencing error. The rate of sequencing error per nucleotide was estimated by measuring the number of nucleotide differences between segments assembled from repeated runs of the same influenza samples from a separate project (61 samples, 142 lanes in total; some samples had been sequenced more than twice).

The resultant expected number of differences was small, corresponding to a mean number of differences of 1.1 nucleotides across a whole genome (standard deviation: 2.4nt).

Finally, we combined these rates to generate a cut-off for each pair of samples above which we did not consider samples to be linked, using the difference in sampling dates to determine the number of substitutions we would expect to see as a consequence of viral mutation. Using 95% confidence intervals, this resulted in cut-offs of between 5.8-7.3nt and 5.8-6.2nt between pairs of samples when using the literature-estimated or BEAST-derived substitution rates, respectively.
